# Genetic Diversity in *Fusarium graminearum* from a Major Wheat-Producing Region of Argentina

**DOI:** 10.3390/toxins3101294

**Published:** 2011-10-20

**Authors:** Cora Lilia Alvarez, Stefania Somma, Robert H. Proctor, Gaetano Stea, Giuseppina Mulè, Antonio F. Logrieco, Virginia Fernandez Pinto, Antonio Moretti

**Affiliations:** 1 Departamento de Biodiversidad y Biología Experimental, Universidad de Buenos Aires, Buenos Aires, Argentina; Email: cora@bg.fcen.uba.ar (C.L.A.); virginia@qo.fcen.uba.ar (V.F.P.); 2 Institute of Sciences of Food Production, CNR, Via Amendola 122/0, 70126 Bari, Italy; Email: stefania.somma@ispa.cnr.it (S.S.); gaetano.stea@ispa.cnr.it (G.S.); giuseppina.mule@ispa.cnr.it (G.M.); antonio.logrieco@ispa.cnr.it (A.F.L.); 3 Bacterial Foodborne Pathogen and Mycology Research Unit, National Center for Agricultural Utilization Research, ARS, USDA, Peoria, IL 61604, USA; Email: robert.proctor@ars.usda.gov

**Keywords:** Fusarium Head Blight, AFLP, translation elongation factor 1−α, β-tubulin, deoxynivalenol, 3-acetyldeoxynivalenol, 15-acetyldeoxynivalenol

## Abstract

The *Fusarium graminearum* species complex (FGSC) is a group of mycotoxigenic fungi that are the primary cause of Fusarium head blight (FHB) of wheat worldwide. The distribution, frequency of occurrence, and genetic diversity of FGSC species in cereal crops in South America is not well understood compared to some regions of Asia, Europe and North America. Therefore, we examined the frequency and genetic diversity of a collection of 183 FGSC isolates recovered from wheat grown during multiple growing seasons and across a large area of eastern Argentina, a major wheat producing region in South America. Sequence analysis of the translation elongation factor 1−α and β-tubulin genes as well as Amplified Fragment Length Polymorphism (AFLP) analyses indicated that all isolates were the FGSC species *F. graminearum sensu stricto*. AFLP analysis resolved at least 11 subgroups, and all the isolates represented different AFLP haplotypes. AFLP profile and geographic origin were not correlated. Previously obtained trichothecene production profiles of the isolates revealed that the 15-acetyldeoxynivalenol chemotype was slightly more frequent than the 3-acetyldeoxynivalenol chemotype among the isolates. These data extend the current understanding of FGSC diversity and provide further evidence that *F. graminearum sensu stricto* is the predominant cause of FHB in the temperate main wheat-growing area of Argentina. Moreover, two isolates of *F. crookwellense* and four of *F. pseudograminearum* were also recovered from wheat samples and sequenced. The results also suggest that, although *F. graminearum sensu stricto* was the only FGSC species recovered in this study, the high level of genetic diversity within this species should be considered in plant breeding efforts and development of other disease management strategies aimed at reducing FHB.

## 1. Introduction

*Fusarium graminearum* (Schwabe) (teleomorph *Gibberella zeae* (Schwein.)) is considered the main cause of Fusarium Head Blight (FHB) worldwide [1]. The disease is of significance because it reduces yield and quality of grains and also results in mycotoxin contamination of wheat kernels, primarily with trichothecenes [2], that have adverse effects on animal and human health [3]. The most common trichothecenes produced by *F. graminearum* are deoxynivalenol (DON), its acetylated derivatives 3-acetyldeoxynivalenol (3ADON) and 15-acetyldeoxynivalenol (15ADON), and nivalenol (NIV), and its acetylated derivative 4-acetyl-nivalenol (4ANIV or fusarenone X) [2]. Three trichothecene production profiles (chemotypes) predominate in this species: the production of NIV and 4ANIV (NIV chemotype); the production of DON and 3ADON (3ADON chemotype); and the production of DON and 15ADON (15ADON chemotype) [4]. Surveys of trichothecenes in wheat grains infected with *F. graminearum* indicate that the 3ADON and 15ADON chemotypes predominate in North America, South America and Europe. The NIV chemotype is more common in Europe than America [5,6], however, NIV strains have recently been found in North America [7]. Previous reports from Argentinean strains of *F. graminearum* isolated from wheat have shown controversial results: Ramirez *et al.* [8] reported that all the strains produced DON and a few of them produced 3ADON; Lori *et al.* [9] reported that the strains produced DON, 3ADON and NIV; finally Fernandez Pinto *et al.* [10] reported the production of DON, 3ADON, NIV and mainly 15ADON. A recent report of the mycotoxin production in *F. graminearum* strains from different regions in the great production area of wheat in Argentina has shown that strains producing DON and 15ADON are as frequent as those producing DON and both acetylated derivatives, 3ADON and 15ADON. In addition, NIV and DON were produced simultaneously by 23.6% of the isolates, although DON was always produced in higher quantities in these isolates [11]. The co-occurrence of DON and NIV in the same isolates is surprising since the genetic basis of DON and NIV chemotypes has been established to be due to differences in *TRI13* and *TRI7* [12]. These genes are non-functional in DON-producing isolates so it is unclear how DON and NIV could both be produced by a single isolate. Similarly, the co-occurrence of 3ADON and 15ADON in the same isolates is surprising since the genetic basis of 3ADON and 15ADON chemotypes is due to different forms of *TRI8* in 3ADON and 15ADON-producing strains [13].

Recognition by morphological characters sometimes is not enough for accurate identification of fungal isolates at the species level. In the last decade, genealogical concordance phylogenetic species recognition (GCPSR) was used to investigate genetic diversity and species limits within *F. graminearum* species complex (FGSC) [14]. Based on DNA sequences of 13 genes, FGSC was resolved in 13 species: *Fusarium acacia-mearnsii*, *Fusarium aethiopicum*, *Fusarium asiaticum*, *Fusarium austroamericanum*, *Fusarium boothii*, *Fusarium brasilicum*, *Fusarium cortaderiae*, *Fusarium gerlachii*, *F. graminearum sensu stricto*, *Fusarium meridionale*, *Fusarium mesoamericanum*, *Fusarium ussurianum,* and *Fusarium vorosii* [15,16,17,18]. Therefore, a correct identification of FGSC species is a critical point in order to correctly predict and evaluate the potential mycotoxigenic risk related to the occurrence of each species on wheat. The most geographically widespread species of the FGSC is *F. graminearum sensu stricto*, which predominates on wheat in North and South America and in Europe [19,20,21,22]. On other hand, a recent report from South America has shown the occurrence on wheat also of another species of FGSC, *F. meridionale* [23]. 

**Figure 1:**
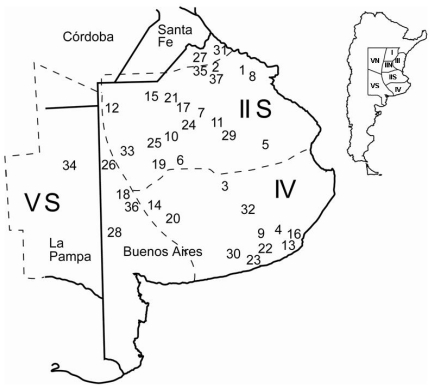
Map showing the sample fields in the main wheat cultivation area in Argentina. Arabic numerals represent the locations of the 37 fields from which wheat was harvested and isolates were recovered. Wheat cropping area is distributed according to agrometeorological conditions into five regions (I to V, with region II and V divided in two subregions named North (N) and South (S)).

In Argentina, wheat is cultivated in a 6,000,000 ha area that is divided into five regions (designated I to V, Figure 1) according to agrometeorological conditions. 

Regions II and V are further divided into North (N) and South (S) based on differing climatic conditions in each. The regions/sub-regions IIS, IV and VS are considered the main production area because in these regions the production area is the widest in Argentina, the wheat cultivation is more intense and yields are greatest. Sub-region IIS covers almost the entire northern half of the province of Buenos Aires and the extreme South of the province of Santa Fe; region IV covers the south east of the province of Buenos Aires; and sub-region VS covers the far east of the province of La Pampa and the east and south east of the province of Buenos Aires. The temperature and humidity decrease along subregions IIS, IV and VS, with more extreme conditions in region VS [24]. Within these extended areas, where FHB epidemics occur often due mainly to favourable weather conditions [25], *F. graminearum sensu stricto* has been reported to be the main cause of FHB only in two localities of Buenos Aires province [20]. However, the results, reported by Sampietro *et al.* [26], demonstrated that the *F. graminearum* complex diversity in Argentina is greater than previously assessed since *F. meridionale* and *F. boothi* were isolated at a high concentration from maize grains cultivated in a wide area in northwest Argentina. 

The main objectives of this study were:

- deepen and broaden the genetic characterization of strains of the FGSC isolated in multiple seasons from wheat kernels in a wider wheat cropping area from Buenos Aires Province in Argentina than previously studied, by using Amplified Fragment Length Polymorphism (AFLP) and sequence analyses of translation elongation factor 1−α (*TEF1*) and β-tubulin (*TUB2*) genes;
- evaluate the genetic variability of a wide population of FGSC by using AFLP;
- assess the main FGSC species occurring on wheat in Argentina, comparing it to the contradictory data previously reported from different cereals.

## 2. Material and Methods

### 2.1. Isolation and Identification

Thirty-seven wheat samples from the main wheat production area of Argentina were analyzed. Each sample was from different localities selected over all the Buenos Aires province area and randomly collected from wheat harvested in 2001, 2003 and 2004 (Figure 1). Samples were at least 1 kg in size and not sorted by grain quality (all grades included). For the isolation of the internal mycoflora, 100 kernels from each sample were surface disinfested in a 10% sodium hypochlorite for 1 minute, rinsed in sterile distilled water, then in 70% ethyl alcohol, and dried on a filter paper. The kernels were plated on a *Fusarium*-selective medium composed of potato dextrose agar (PDA) containing 0.2 g/L of pentachloronitrobenzene (PCNB) and incubated for 5 days at 25 °C under fluorescent light (12 h photoperiod) [27]. Putative *Fusarium* isolates recovered from the wheat kernels were purified by single-spore isolation. Morphological identification was determined with cultures grown on the low nutrient medium SNA (Spezieller Nahrstoffarmer Agar) with filter paper as described by Leslie and Summerell [27]. 

The 189 fungal isolates used in this study are listed in Table 1 together with data on their geographic origin and the year in which the corresponding wheat samples were harvested. 

**Table 1:** Locality and year of isolation of 189 *Fusarium* strains from Buenos Aires regions of Argentina used in this study.

Sample Fields *	Geographical Region	Year		Strains ITEM Number **
27-Pergamino	IIN	2001		8544-8545-8546-8547-8548
2-Arrecifes	IIS	2001		8310-8311
12-Coronel Villegas	IIS	2001		8386-8387
24-Nueve de Julio	IIS	2001		8467-8468-8471-8473-8475-8476-8477-8478-8479-8480-8481-8482-8483-8485-8486-8487-8488-8492-8493-8497-8502-8504-8505-8510-8512-8513-8514-8516-8517-8526-8531-8538
29-Saladillo	IIS	2001		8552
31-San Pedro	IIS	2001		8559-8560-8561-8562-8563
35-Salto	IIS	2001		8602-8603-8604
5-Belgrano	IIS	2003		8325-8327-8329-8330-8334-8335
8-Cardales	IIS	2003		8339-8340-8341
25-Pehuajo	IIS	2003		8540
1-Areco	IIS	2004		8308
6-Bolivar	IIS	2004		8337
7-Bragado	IIS	2004		8338
10-Carlos Casares	IIS	2004		8357-8361-8362-8363
11-25 de Mayo	IIS	2004		8364-8365-8366-8367-8368-8369-8370-8371-8372-8373-8374-8375-8376-8377-8379-8380-8381-8382-8589
15-General Pinto	IIS	2004		8412-8413
17-General Viamonte	IIS	2004		8417
19-H Yrigoyen	IIS	2004		8420
21-Lincoln	IIS	2004		8425-8426
33-Trenque Lauquen	IIS	2004		8583-8585
37-Suipacha	IIS	2004		8564 ***
16-General Pueyrredon	IV	2001		8414-8415-8416
22-Loberia	IV	2003		8427-8428-8429-8430-8432-8433
3-Azul	IV	2004		8313-8314-8315-8316-8318-8319-8320-8321
4-Balcarce	IV	2004		8322-8323-8324
9-Cardenau	IV	2004		8342-8343-8344-8345-8346-8347-8348-8349-8350-8351-8352-8353-8354-8355-8356
13-General Alvarado	IV	2004		8388-8393-8394-8395-8396-8397-8398-8399-8401-8402-8404-8405-8406-8407
14-General Lamadrid	IV	2004		8408-8409-8410
20-Laprida	IV	2004		8421-8422-8423-8424
23-Necochea	IV	2004		8435-8436-8438-8439-8440-8441-8444-8446-8447-8448-8453-8454-8456-8458-8460-8461-8462-8463-8464
30-San Cayetano	IV	2004		8555-8556-8558
32-Tandil	IV	2004		8574-8576-8581-8582-8566 ***-8567 ***-8569 ***
36-Coronel Suarez	VS	2003	8383 ****-8384 ****	
18-Guamini	VS	2004	8418-8419	
26-Pellegrini	VS	2004	8542	
28-Saavedra	VS	2004	8549	
34-Conhello	VS	2004	8591-8600-8601	

* The numbers show the location of the sample fields in Figure 1; ** Reference number of the Fungal Collection of Institute of Sciences of Food Production, Research National Council (ISPA-CNR), Bari, Italy; *** *F. pseudograminearum*; **** *F. crookwellense*.

To aid in FGSC species identifications, we employed the following reference strains used as out-group in AFLP analyses, which were kindly provided by D. Geiser (The Penn State University): NRRL 2903 for *F. austroamericanum* (syn. Lineage 1); NRRL 26916 for *F. boothi* (syn. Lineage 3); NRRL 25797 for *F. mesoamericanum* (syn. Lineage 4); NRRL 26754 for *F. acaciae-mearnsii* (syn. Lineage 5); NRRL 13818 for *F. asiaticum* (syn. Lineage 6); NRRL 28336 and NRRL 5883 for *F. graminearum* (syn. Lineage 7); NRRL 29297 for *F. cortaderiae* (syn. Lineage 8) [14,15]. All strains were deposited in the culture collection of the Institute of Sciences of Food Production, Bari, Italy (ITEM) [28]. All 189 strains from wheat kernels in Argentina (53 isolated in 2001, 18 in 2003, and 118 strains isolated in 2004) were subjected to gene sequencing and AFLP analysis.

### 2.2. DNA Extraction

Fungal isolates were grown for 48 h in shake cultures (125 rpm) at 25 °C in 100 mL of Wikerham’s medium (4% D-glucose, 0.5% peptone, 0.3% yeast extract, 0.3% malt extract). The resulting mycelium was vacuum-filtered on Whatman N° 4 filter paper, washed with distilled water, frozen, and lyophilised. About 25-30 mg of lyophilised mycelium was ground and used to prepare genomic DNA with the E.Z.N.A. Fungal DNA Miniprep Kit (Omega Bio-tek, Doraville, GA). Extracted DNA was dissolved in 100 μL of sterile water and stored at −20 °C. DNA concentrations were estimated by comparison with a known concentration of a DNA ladder following agarose gel electrophoresis.

### 2.3. Mating Type Analysis

A multiplex PCR was used to detect the mating type genes of the isolates. The primers used to amplify the Mating type 1 (MAT1-1) idiomorph were M12-1: 5'-ATGGATACCTCCTTCAGTTTC-3' and M12-2: 5'-GATCATCCGGCTCCTCAGGGT C-3'; and the primers used to amplify the Mating type 2 (MAT1-2) idiomorph were M21-1: 5'-ATGAGCACCCTTATGTTGATG-3' and M21-2: 5'-TCAGACGTTGTTTTTGCTGAGC-3' [14]. PCR was performed in a final volume of 20 μL, using 5 ng of DNA, 0.3 μL of each primer (10 mM), 0.4 μL of dNTPs (10 mM) and 0.1 μL of Taq DNA Polymerase (Hot Master, 5 U/μL). The thermal cycler parameters were: initial denaturation for 5 min at 94 °C; 40 cycles of 35 s at 94 °C, 55 s at 54 °C, 2 min at 72 °C; and a final extension for 7 min at 72 °C. The expected amplicon sizes were 1500 bp and 800 bp for MAT1-1 and MAT1-2, respectively. 

### 2.4. DNA Sequencing

Sequence analysis of *TEF1* and *TUB2* genes employed PCR conditions and primers described by O’Donnell *et al.*  [14]. PCR products were analyzed by agarose gel electrophoresis to confirm that a ~700 bp fragment was amplified for *TEF1* and a ~1300 bp fragment was amplified for *TUB2*. Fragments were purified by filtration through Shepadex G-50 (Sigma) prior to DNA sequencing. Sequence analysis was carried out with the Big Dye Terminator Cycle Sequencing Ready Reaction Kit (Applied Biosystems). Sequences were aligned in ClustalW [29] by using default settings with MEGA software [30]. Aligned sequence data were subjected to maximum-parsimony (MP) analysis by using the Close-Neighbor-Interchange algorithm with 10 random-addition replicates with the MEGA 4 software. Clade validation was assessed by bootstrap analysis [31], using heuristic searches with 1000 replicates. Previously generated *TEF1* and *TUB2* sequences obtained from the National Center for Bioechnology Information (NCBI) [32] were also included to allow comparative analysis.

### 2.5. AFLP Analysis

AFLP analysis was performed with the AFLP^TM^ Microbial Fingerprint Kit (Applied Biosystems, Fostercity, CA). Restriction enzymes *EcoRI* and *MseI* (New England Biolabs, Hitchin, Hertfordshire, United Kingdom) were used to digest 10 ng of genomic DNA from each isolate, and DNA fragments were ligated with T4 DNA ligase (New England Biolabs, Hitchin, Hertfordshire, United Kingdom) to double-stranded restriction site-specific adaptors supplied by the kit (AFLP^TM^ Microbial Fingerprint). The selective reactions used three primer combinations: *EcoRI*+AC/*MseI*+CC, *EcoRI*+AT/*MseI*+CG and *EcoRI*+G/*MseI*+CT, where the *EcoRI* primers were labeled with FAM, NED and JOE fluorescent dyes (Applied Biosystems), respectively. After amplification, the products were mixed with deionized formamide and the GeneScan-500 (Rox) size standard (Applied Biosystems), and then separated by capillary electrophoresis on ABI PRISM 310 Genetic Analyzer. Raw data were analyzed using GeneScan Collection software (version 3.1.2, Applied Biosystems), and AFLP patterns were analyzed with Genotyper software (Applied Biosystems). AFLP peaks ranging from 150 to 500 bp in length and with peak height limit of at least 200. Bands of the same size in different samples were assumed to be homologous and to represent the same allele. Bands of different sizes were considered independent loci with two alleles (present and absent). AFLP data of 183 *Fusarium* Argentinean isolates and 8 reference strains of the FGSC were exported in a binary matrix, in which “1” represented the presence of the peak and “0” its absence. Statistical software NTSYS (version 2.02, Exeter software, Setauket, NY) was used for cluster analysis by using DICE similarity coefficient and the un-weighted pair group method (UPGMA). The trees were generated by using the sequential agglomerative hierarchical nested clustering program (SAHN; Exeter software, Setauket, NY). Each DNA sample was subjected to AFLP analysis in at least two independent experiments and yielded the same AFLP profile each time it was run. Strains with >98 AFLP band similarity were considered to belong to the same haplotype.

## 3. Results

### 3.1. Strain Isolation and Identification

One hundred eighty-nine isolates of *Fusarium* were recovered from wheat kernels that were harvested from across the major wheat-producing region of Argentina during the 2001, 2003 and 2004 growing seasons. All isolates were subjected to PCR to analyze for the presence of the *MAT-1* and *MAT-2* idiomorphs. The PCR yielded amplicons for both idiomorphs from 183 isolates and an amplicon for only the *MAT-2* idiomorph from the remaining six isolates (ITEM 8364, 8366, 8367, 8369, 8383 and 8384). To identify the species to which each isolate belongs, fragments of the *TEF1* and *TUB2* genes were also amplified and sequenced and then subjected to BLAST analysis against the Fusarium-ID database [33]. This analysis indicated that the isolates with *MAT-2* only were either *F. crookwellense* (ITEM 8383 and 8384) or *F. pseudograminearum* (ITEM 8364, 8366, 8367 and 8369), whereas isolates with both idiomorphs were *F. graminearum sensu stricto* (Table 1). 

### 3.2. DNA Sequence-Base Phylogenetic Analysis

**Figure 2:**
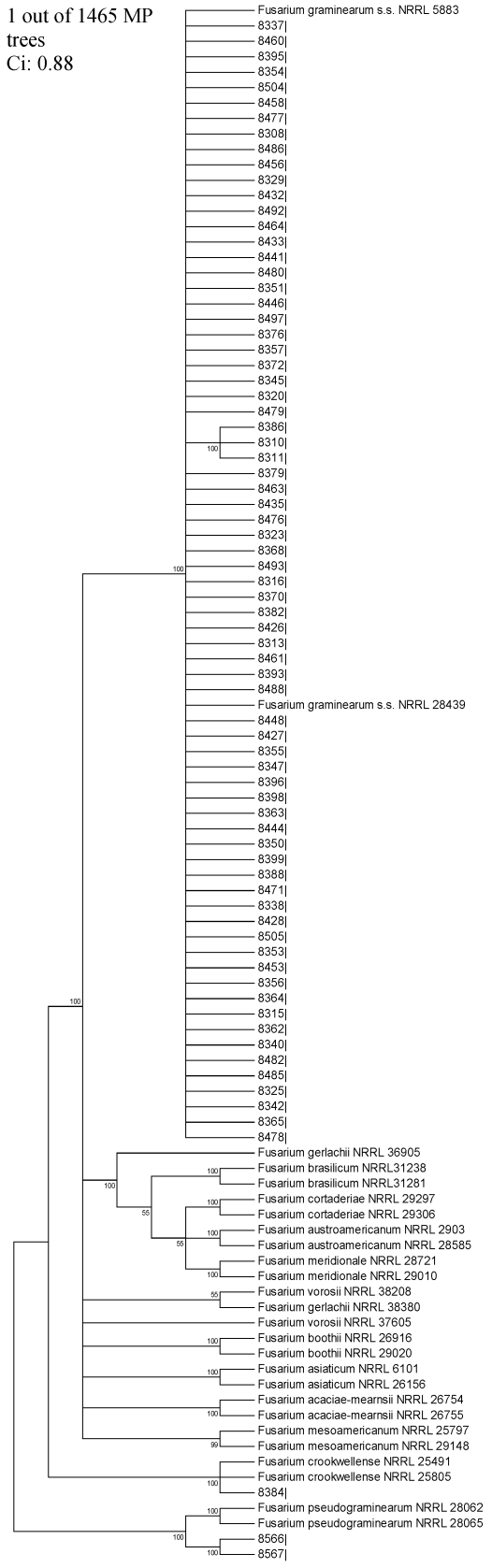
One of the 1465 most-parsimonious phylograms inferred from translation elongation factor gene sequences. The phylogenetic tree is composed by 72 randomly selected sequences of *F. graminearum* Argentinean strains, 2 of *F. pseudograminearum*, 1 of *F. croockwellense* and the strains used to define species inside FGSC [15,17], combined with sequences of the eleven species of the FGSC complex [17] registered in NCBI database. Branches that received >50% bootstrap values were indicated.

The *TEF1* and *TUB2* sequences from a subset of isolates were also subjected to phylogenetic analysis. This analysis employed sequences from 72 randomly selected isolates identified as *F. graminearum sensu stricto* (Table 1) as well as two isolates identified as *F. pseudograminearum* (ITEM 8366 and 8367), and one identified as *F. crookwellense* (ITEM 8384). In addition, as reference, sequences for FGSC species were downloaded from NCBI and included in the analysis [13,15]. One-thousand-four-hundred-sixty-five phylogenetic trees were formed with *TEF1* (Consistency index, Ci: 0.88 and Retention Index, Ri: 0.96) (Figure 2). Similar phylogram was obtained with *TUB2* sequences. The 72 *F. graminearum* wheat isolates from Argentina formed a distinct clade with reference strains of *F. graminearum sensu stricto* that was distinct from the other FGSC species included in the analysis. These results further support the identity of the majority of isolates from Argentinean wheat as *F. graminearum sensu stricto*.

### 3.3. Nucleotide Sequence Accession Numbers

DNA sequence data generated in the present study for phylogenetic analysis belonging to the 75 strains of *F. graminearum sensu stricto* (72), *F. pseudograminearum* (2), and *F. crookwellense* (1) have been deposited in GenBank under accession numbers from JN687849 to JN687923.

### 3.4. AFLP Analysis

**Figure 3:**
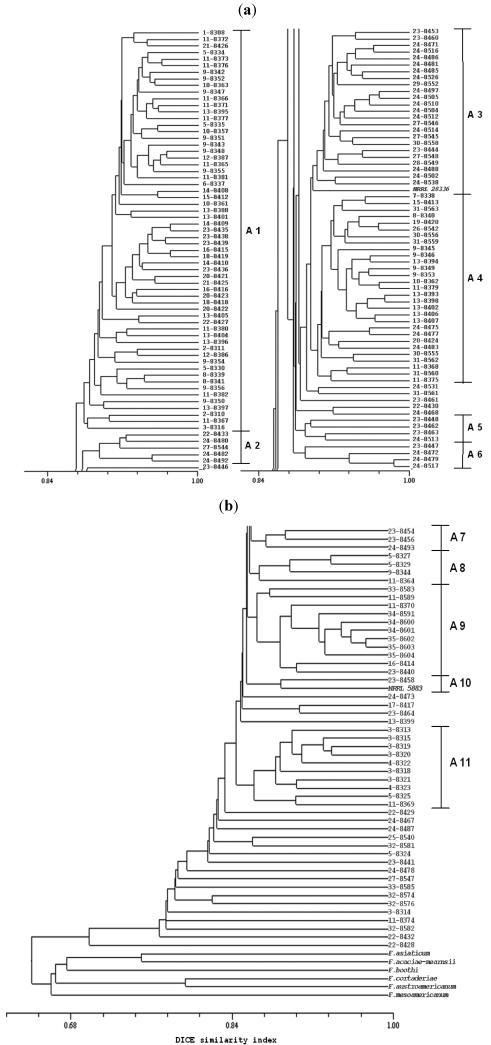
Dendrogram generated with AFLP data of 183 *F. graminearum* Argentinean strains and *Fusarium graminearum* complex standard isolates, generated by NTSYS software with DICE similarity index. Strains are signed with the ITEM number preceded by the field number. Subclusters from A1 to A11 are reported in the figure.

Isolates identified as *F. graminearum sensu stricto* were also subjected to AFLP analysis. The AFLP protocol generated a total of 232 reproducible loci ranging in size from 150 to 500 bp; 57 loci were generated with the *EcoRI*-AC/*MseI*-CC primer combination, 52 loci with the *EcoRI*-AT/*MseI*-CG combination, and 123 loci with the *EcoRI*-G/*MseI*-CT combination. Twelve loci were uninformative and were excluded from the UPGMA analysis and determination of DICE similarity coefficient. Based on the UPGMA-generated dendrogram, the isolates of *F. graminearum sensu stricto* isolated from wheat as well as the reference strains for this species formed a cluster that was distinct from the other six FGSC species included in the analysis (Figure 3a,b). 

The analysis also indicated that 152 (83%) of the *F. graminearum sensu stricto* isolates were highly similar to one another and to reference strains NRRL 28336 and NRRL 5883 for this species; the DICE similarity coefficients for the 152 isolates and two reference strains were >0.85 (Figure 3a,b). Although Argentinean isolates were grouped in one main cluster, the dendrogram indicates that most of isolates could be grouped in at least 11 sub-clusters, named in Figure 3a,b from A1 to A11. Among them, 10 isolates branched as a single member. A clear cluster made of 10 strains, mainly isolated from the fields, numbers 3 and 4 of the IV region, was formed. The resolved sub-clusters were not correlated with geographical origin, year of harvest or chemotype. No isolate with the same haplotype was found; therefore no isolate was a clonal one. The other 17 isolates of *F. graminearum sensu stricto* analyzed were less similar to one another as well as the other 152 isolates; they exhibited similarity coefficients of <0.84. In particular, among this group of strains, ITEM 8428 branched at a similarity value of 0.70, being the most distant from all the other strains (DICE similarity coefficient >0.77). This relatively low level of similarity among the 17 isolates was comparable to levels of similarity among some FGSC species; *i.e*., the DICE similarity coefficient for *F. austroamericanum versus F. cortaderiae* and for *F. acaciae-mearnsii versus F. asiaticum* were ~0.79 and ~0.75 respectively. 

## 4. Discussion

Previous studies on the genetic diversity of *F. graminearum sensu stricto* from wheat in Argentina have been limited. In one study [20], the genetic diversity of 113 isolates recovered from wheat from two locations and during one growing season was examined by AFLP analysis. However, the profile of trichothecene production was not determined. In a second study [11], trichothecene production profiles were examined for 144 isolates from multiple locations across a large area of the major wheat-producing region of Argentina. However, the genetic diversity of the strains was not examined. In the current study, we examined the species identity and genetic diversity (via AFLP analysis) of the collection of 144 isolates noted above, plus 39 additional isolates, for which trichothecene production was previously determined [11]. Our analysis provides further evidence that *F. graminearum sensu stricto* is the predominant species of the FGSC in the temperate wheat production region of Argentina. This is the same species that predominates in North America and Europe [15,21,22,34,35] and that has been reported elsewhere in South America [20,23]. However, many reports have also shown that other FGSC species can often co-occur with *F. graminearum sensu stricto* in the same geographical areas, as it was reported in New Zealand and China [36,37]. Recent reports [26] indicated that the FGSC species *F. meridionale* and *F. bothii* predominate on maize in north western, subtropical Argentina, whereas *F. graminearum sensu stricto* predominates on maize and wheat in the temperate regions of Argentina. The co-occurrence of potentially interfertile but phylogenetically distinct FGSC species could promote interbreeding between the species and, therefore, formation of new genotypes that could affect pathogenicity, host range, or toxin production. The homogeneity of the Argentinean population of FGSC from wheat reported here and previously [20] could provide an advantage with respect to disease management, plant breeding strategies and quarantine regulations. However, the occurrence of other FGSC species in Argentina should be verified by analyzing other potential host plants, such as maize, barley, and other small cereals. In particular, the occurrence of *F. cortaderiae*, considered by O’Donnell *et al.* [15] to have a South American origin, should be evaluated.

From the preliminary AFLP analysis, the high number of polymorphic loci estimated within the 183 field isolates of *F. graminearum sensu stricto* from Argentina, indicate that the genetic diversity within this species is relatively high. Moreover, the high percentage of haplotypes occurring within the field population of *F. graminearum* highlighted a high degree of genotypic diversity. In particular, the occurrence of two strains, ITEM 8432 and 8428, provided of a low level of similarity with respect to the whole set of strains, due to both unique loci and relatively unique combinations of some loci, increased the genetic/genotypic diversity of the population here investigated. However, these data agree with the data shown by Ramirez *et al.* [20] that found a high level of genetic exchange between two populations of *F. graminearum sensu stricto* isolated from two different localities in Argentina and considered them potentially part of a larger randomly mating population. Moreover, this genotypic variability could be explained by out-crossing, which appears to be common in *F. graminearum* in South and North America [8,22,38] and by gene flow, since the potential long distance dispersal of spores of *F. graminearum* has been well documented [39]. In this study, we utilized two different approaches, AFLP analysis and analysis of DNA sequence of two genes, to gain insight into evolutionary relationships within a population of FGSC isolated from wheat in Argentina. Phylogenetic analysis based on comparisons of *TEF1* and *TUB2* sequences assigned all strains analyzed to *F. graminaerum sensu stricto*. The sequence data are consistent with AFLP data that grouped the Argentinean isolates with the two *ex type* strains of *F. graminearum sensu stricto* and delineated them from *ex-type* strains of six other FGSC species. Our AFLP data indicate that the level of diversity among some *F. graminearum sensu stricto* isolates was greater than the level of diversity between the two FGSC species *F. austroamaricanum* and *F. cortaderiae* (Figure 3a,b). However, since phenetic criteria such as percent identity of AFLP are considered less reliable and biologically meaningful than GCPSR [15], these data must be evaluated carefully. 

The mycotoxin production of 115 out of the 183 strains, was the object of a previous study [11] that showed that DON was produced by most of the isolates. Isolates producing 15ADON were more common than those producing 3ADON. It is interesting to underline that both 3ADON and 15ADON strains of *F. graminearum sensu stricto* here reported do not seem to be genetically distinct entities since they occur along the entire length of the AFLP-generated dendrogram. Although this is in contrast with O’Donnell *et al.* [16] that showed 3ADON and 15ADON isolates of *F. graminearum sensu stricto* from USA as genetically distinct populations, our data agree with previous studies based on AFLP that could not resolve into separate clades 3ADON and 15ADON isolates [20,21,39].

## 5. Conclusions

This report contributes to an improved understanding of the genetic diversity within *F. graminearum sensu stricto* across an extended wheat-cropping area of Argentina. This information will be useful for monitoring any future changes in the pathogen related to trichothecene production and for adopting strategies to control Fusarium Head Blight in Argentina. Finally, although only a few strains were isolated, our results provide the first report of *F. pseudograminearum* and *F. crookwellense* on wheat in Argentina.
